# Whole-exome identifies germline variants in families with obstructive sleep apnea syndrome

**DOI:** 10.3389/fgene.2023.1137817

**Published:** 2023-05-09

**Authors:** Pedro Guimarães de Azevedo, Maria de Lourdes Rabelo Guimarães, Anna Luiza Braga Albuquerque, Rayane Benfica Alves, Bianca Gomes Fernandes, Flavia Marques de Melo, Raony Guimaraes Corrêa Do Carmo Lisboa Cardenas, Eitan Friedman, Luiz De Marco, Luciana Bastos-Rodrigues

**Affiliations:** ^1^ Centro de Tecnologia em Medicina Molecular, Faculdade de Medicina, Universidade Federal de Minas Gerais, Belo Horizonte, Brazil; ^2^ The Preventive Personalized Medicine Center, Assuta Medical Center and the Sackler School of Medicine, Tel-Aviv University, Tel Aviv, Israel; ^3^ Department of Surgery, Universidade Federal de Minas Gerais, Belo Horizonte, Brazil; ^4^ Department of Nutrition, Universidade Federal de Minas Gerais, Belo Horizonte, Brazil

**Keywords:** whole exome, sequencing, obstructive sleep apnea syndrome, OSAS, candidate genes

## Abstract

**Background:** Obstructive sleep apnea syndrome (OSAS) (OMIM #107650) is characterized by complete or partial obstruction of the upper airways, resulting in periods of sleep associated apnea. OSAS increases morbidity and mortality risk from cardiovascular and cerebrovascular diseases. While heritability of OSAS is estimated at ∼40%, the precise underlying genes remain elusive. Brazilian families with OSAS that follows as seemingly autosomal dominant inheritance pattern were recruited.

**Methods:** The study included nine individuals from two Brazilian families displaying a seemingly autosomal dominant inheritance pattern of OSAS. Whole exome sequencing of germline DNA were analyzed using Mendel, MD software. Variants selected were analyzed using Varstation^®^ with subsequent analyses that included validation by Sanger sequencing, pathogenic score assessment by ACMG criteria, co-segregation analyses (when possible) allele frequency, tissue expression patterns, pathway analyses, effect on protein folding modeling using Swiss-Model and RaptorX.

**Results**: Two families (six affected patients and three unaffected controls) were analyzed. A comprehensive multistep analysis yielded variants in *COX20* (*rs946982087*) (family A), *PTPDC1* (*rs61743388*) and *TMOD4* (*rs141507115*) (family B) that seemed to be strong candidate genes for being OSAS associated genes in these families.

**Conclusion**: Sequence variants in *COX20*, *PTPDC1* and *TMOD4* seemingly are associated with OSAS phenotype in these families. Further studies in more, ethnically diverse families and non-familial OSAS cases are needed to better define the role of these variants as contributors to OSAS phenotype.

## Introduction

Obstructive sleep apnea syndrome (OSAS) (OMIM #107650) affects 936 million adults worldwide (Benjafield et al., 2019) and is hallmarked by the interruption of or a significant decrease in airflow in the presence of respiratory effort during sleep, leading to increased sleepiness and significantly increases cardiovascular morbidity and mortality risks (Lévy et al., 2015). While the precise etiological factors for OSAS are not clear, disturbances in ventilatory control, craniofacial anatomy, and being overweight, as well as genetic factors are major contributors to OSAS (Peppard and Hagen, 2018).

Familial clusters of OSAS have been reported and in fact heritability and genetic factors have been estimated to contribute ∼40% to OSAS phenotype (Redline et al., 1995; Patel et al., 2008). Over the past few years, sequence variants in candidate genes seemingly associated with OSAS (as objectively quantified by AHI [(apnea–hypopnea index (AHI), defined as the number of apnea and hypopnea events per hour of sleep (American Academy of Sleep Medicine, 2014) (International Classification of Sleep Disorders, 2014) have been reported (Larkin et al., 2010), as well as variants identified in the course of admixture mapping (Wang et al., 2019) and genome wide association studies (GWAS) (Cade et al., 2021). However, few genes have been reported and validated as “OSAS genes” using these approaches (Mohit, Shrivastava, Chand, 2021). The aim of the current study was to identify pathogenic germline sequence variants that could be associated with predisposition and/or development of OSAS using whole-exome DNA sequencing of individuals from two Brazilian OSAS families.

## Subjects and methods

**FIGURE 1:**
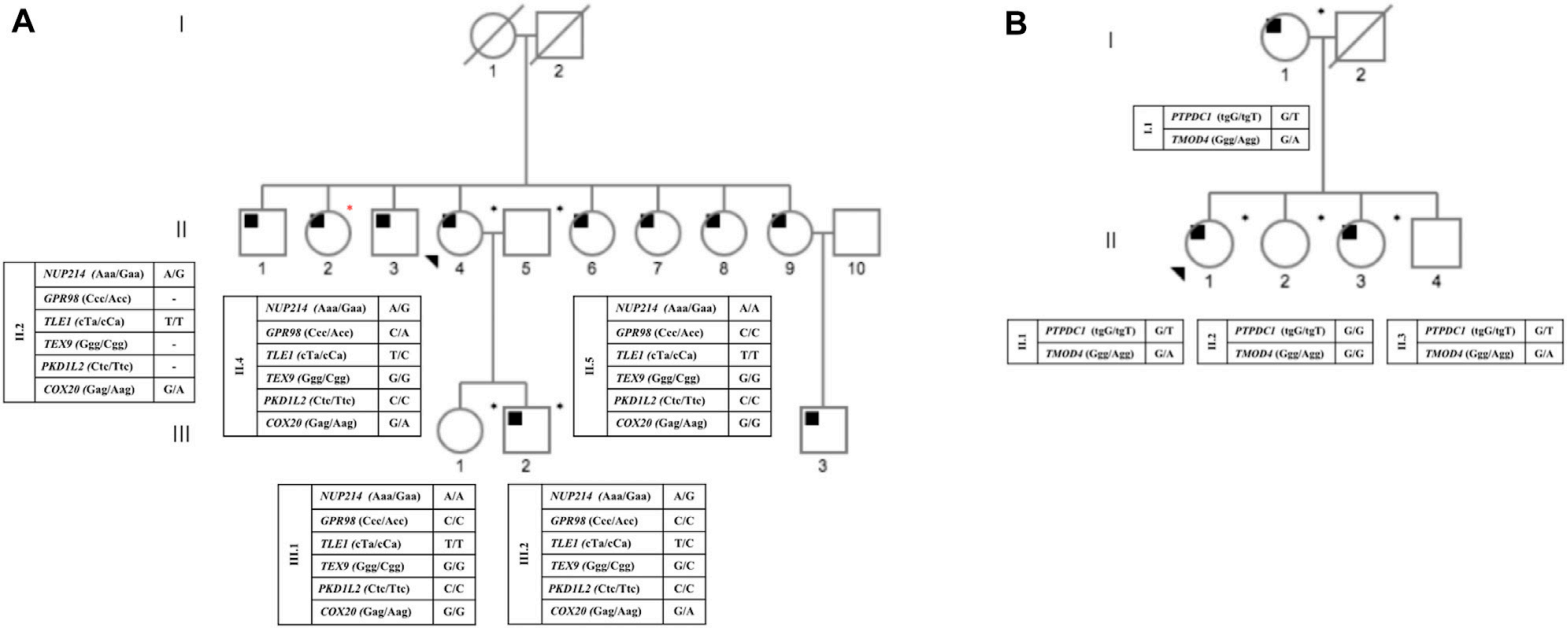
Pedigree status from families A and B, with respect to OSA and the genotypes for each identified heterozygous mutation. Circles and squares represent female and male members, respectively. The proband is indicated by a black arrow. Deceased members are shown by diagonal lines. Black squares represent affected individuals; blank eigenforms indicate unaffected individuals; *DNA from individuals submitted to exome analysis; *individual who participated only in the segregation study. **(A)** pedigree from family A; **(B)** pedigree from family B.

The study encompassed individuals from two Brazilian families displaying OSAS in at least two generations (Figure 1). These families were referred to the School of Medicine, Universidade Federal de Minas Gerais. They underwent the OSAS evaluation protocol as previously described (Kapur et al., 2017). All OSAS diagnoses were based on the established criteria as specified by the American Academy of Sleep Medicine (2014) (International Classification of Sleep Disorders, 2014). Briefly, each participant underwent complete OSAS-focused clinical evaluation and polysomnography. The inclusion criteria were included: diagnosis of OSAS as defined by the American Academy of Sleep Medicine criteria (2014) and absence of craniofacial dysmorphism, genetic syndromes with OSAS as part of the spectrum of manifestations, drug and alcohol abuse, psychiatric disorders, and dementia; age 18–85 years. Participants with upper airway resistance syndrome (UARS) and central sleep apnea were excluded. In addition, dental data were collected such as the presence of self-reported bruxism, pain in masticatory muscles, noise upon movement of the temporomandibular joint (TMJ), tongue size, floor of mouth (i.e., sublingual space) U-shaped region, bordered inferiorly by the mylohyoid muscle, laterally by the gingiva overlying the lingual surface of the mandible, superiorly by the oral tongue, and posteriorly at the insertion of the anterior tonsillar pillar into the tongue. Genomic DNA was extracted from peripheral blood samples of participants using standard protocols (Lahiri and Nurnberger, 1991). The quality and quantity of each DNA sample were tested by NanoDrop ND -2000 UV-Vis Spectrophotometer (Thermofisher, Waltham, MA). The experimental protocols were approved by the Institutional Review Board at the Universidade Federal de Minas Gerais (CEP UFMG 2.980.453). All participants signed an informed consent.

### Whole-exome sequencing

DNA was subjected to whole exome capturing and sequencing using the Roche NimbleGen V2 chip (Madison, WI) or Nextera (San Diego, CA) with the Illumina HiSeq2000 sequencing platform (San Diego, CA) (Horn, 2012). Sanger sequencing was used to validate all pathogenic variants identified via WES.

For each studied sample, raw sequence files were prepared using the Genome Analysis Tool Kit (GATK). Each fastq file was aligned against the human hg19/GRCh37 reference genome and a variant call format (VCF) file generated for each sample. PCR duplicates were removed using Picard (http://broadinstitute.github.io/picard/), reads around known and detected indels were realigned, and base quality was recalibrated using GATK.

VCF files were analyzed using two different tools: *Mendel, MD* (available at https://mendelmd.org/) (Cardenas et al., 2017), and Varstation^®^ (available at https://varstation.com/). Only variants selected by both software and excluding those common to cases and controls were further analyzed according to the ACMG Standards and guidelines (Richards et al., 2015), using published data and the VarSome platform (https://varsome.com). Genes that harbored “pathogenic”, “likely pathogenic” or “uncertain significance”, were queried for potential relevance to OSA pathogenesis by applying the following criteria: i) predicted functional consequences using the Ensembl database; ii) publications relating each gene to cancer, based on PUBMED search of the gene name; iii) pathway annotation, which includes all pathways in which a given gene product has been involved at Genecards, OMIM, and UniProt databases; and iv) minor allele frequency of less than 1% in gnomAD database. After this analyze, seven variants in genes in Family A and in eight variants in Family B, classified as “Likely benign” or “Benign”, were excluded (Supplementary Tables S1, 2). After these steps of filtering and validating candidate pathogenic variants by Sanger sequencing, we generated a final list of candidate genes (Supplementary Figure S1).

In addition, allele frequencies of all candidate variants were queried with data derived from The Genome Aggregation Database (gnomAD; https://gnomad.broadinstitute.org/; accessed 2nd August 2022) as well as the AbraOM: Online Archive of Brazilian Mutations database (https://abraom.ib.usp.br/; accessed 15th August 2022).

### Structural effect prediction

Wild-type protein models were established according to protein sequence available at UniProt (https://www.uniprot.org/blast/). To obtain model of proteins predicted to be derived from the specific gene variants, Swiss-Model and RaptorX (http://raptosx.uchicago.edu) were used.

## Results

### Clinical data

The study included nine individuals from two Brazilian families displaying a seemingly autosomal dominant inheritance pattern of OSAS (Figure 1). The average age and BMI of family A was 55.25 years old and 23.36 kg/m^2^, respectively, of family B the average age and BMI was 62.75 years old and 30.72 kg/m^2^. Table 1 summarizes all relevant clinical and laboratory data.

**TABLE 1:** Clinical and laboratory data of the individuals studied.

Patient	BMI (kg/m^2^)	Age (Years)	AHI (ev./h)	Min Sat. (%)	Mean Sat (%)	Time below 90%	Desat index (%)	Arousal	CPK	Myogl (mcg/L)	Snore	Nose Brea	Palate	*Mallampati*	Malocclusion (Class)
Family A															
II.2	22.6	73	19.8	79.0	85.0	6.8	13.1	18.6	46.0	13.5	Present	Satisf	Ogival	IV	I
II.4	25.1	71	13.2	82.0	88.0	6.5	16.9	22.9	78.0	18.0	Present	Satisf	Ogival	IV	II
II.5	22.2	70	4.6	92.0	96.0	0.0	4.3	3.2	na	na	Present	Satisf	Ogival	IV	I
III.1	20.7	41	0.4	91.0	96.0	0.0	0.4	0.8	33.0	2.8	Present	Satisf	Ogival	IV	III
III.2	25.5	39	22.9	80.0	86.0	7.0	18.2	0.0	na	na	Present	Satisf	Ogival	IV	I
Family B															
I.1	29.6	86	17.1	88	92	5.8	11.7	24.2	61.3	8.0	Present	Satisf	Ogival	IV	I
II.1	24.5	59	9.9	87	92	4.1	9.0	14.2	57.2	12.5	Present	Satisf	Ogival	IV	II
II.2	35.0	57	4.8	90	95	0.1	3.1	1.8	31.5	5.0	Absent	Satisf	Ogival	IV	I
II.3	33.8	50	23.3	85	89	6.3	13.5	19.6	na	Na	Present	Satisf	Ogival	IV	III
II.4	32.3	48	3.6	91	96	0.8	2.4	1.0	na	na	Absent	Satisf	Ogival	III	I

BMI, body mass index; AHI, Apnea–Hypopnea Index (events/hour); Min Sat. = Minimum Saturation %; Mean Sat. = Mean Saturation%; Desat Index = desaturation index %; CPK, serum creatine phosphokinase (Reference Value: Women: 26–140U/L, Man: 38–174U/L); Miogl = serum myoglobin (Reference Value: <90mcg/L); Nose Brea = nose breathing; Satisf = Satisfactory; na = not available.

### WES and family A analyses

A total of 38,612 common variants in 7,462 genes were detected in two affected individuals of family A (II.4 and III.2). After the initial filtering steps (see Methods) a total of 13 heterozygous sequence variants in 13 genes were selected for further analyses. After ACMG pathogenicity classification, Sanger sequence validation, and co-segregation analyses, only one variant remained as a possible OSAS-related genetic variant (*COX20, rs946982087*) (Supplementary Figure S1A). This variant is not reported in the Brazilian population according to the AbraOM database (https://abraom.ib.usp.br/) or in the GnomAD database (gnomAD; https://gnomad.broadinstitute.org/).

The predicted effect of this sequence variant on protein structure by the Swiss Model/MolProbity (https://swissmodel.expasy.org) is to eliminate the Glu9-Glu11 hydrogen bond [15] thereby destabilizing the protein (Supplementary Figure S2).

### WES and family B analyses

A total of 40,318 sequence variants shared by the three affected individuals of family B (I.1, II.1 and II.3) were present in 6,526 genes. After the initial filtering steps (Supplementary Figure S1B), two missense variants in two genes (*PTPDC1* and *TMOD4*) were selected as candidate genes for validation (Supplementary Figure S1B). The reported frequency of these variants in the Brazilian population (https://abraom.ib.usp.br/) was 0.0017 for *PTPDC1* and 0.00042 for *TMOD4,* respectively. In the GnomAD database the reported frequencies are 0.00046 for *PTPDC1* and 0.00097 for *TMOD4* (https://gnomad.broadinstitute.org/), respectively. No segregation analysis was performed in this family due the lack of available samples.

The pathogenicity of *PTPDC1* variant (*rs61743388*) was investigated. The substitution p.Trp159Cys occurs in the tyrosine-protein phosphatase domain, known to be involved in the control of processes such as cellular division and differentiation and was predicted to be pathogenic according to software that estimates the impact on the biological function of a protein, such as PROVEAN (http://provean.jcvi.org/index.php), MutationTaster and SIFT (https://sift.bii.a-star.edu.sg/) (Supplementary Figure S3).

TMOD4 protein is highly evolutionarily conserved between the amino acids 1–151 and 172–344, encompassing the mutated residue p.Gly323Arg. This missense variant localizes at the beginning of an alpha helix and hence may be deleterious for its function, according to Mutation Taster analysis (Supplementary Figure S4).

## Discussion

In this study, three heterozygous sequence variants that seemingly exert a deleterious effect on protein function in three genes (*COX20*, *PTPDC1*, and *TMOD4*) were identified in two Brazilian OSAS families. COX20 is a nucleus-encoded protein, essential for the assembly of the mitochondrial respiratory chain complex IV (CIV)/cytochrome C oxidase (Szklarczyk et al., 2013). COX20 assists in the stabilization and translocation of newly synthesized COX2 C-terminal into the intermembrane space during CIV assembly and promotes the association between COX2 and metallochaperones SCO1/2, essential for the biogenesis of the cooper core in Complex IV (Lorenzi et al., 2018). Consequently, limiting COX20 levels was associated with unstable, degradable COX2 proteins (Bourens et al., 2014), resulting in impaired complex IV biogenesis and reduced respiratory capacity (Otero et al., 2018). Homozygous *COX2* gene pathogenic variants lead to mitochondrial complex IV deficiency (OMIM# 220110), a disorder clinically hallmarked by neuromuscular findings such as hypotonia, dystonia, and ataxia (Szklarczyk et al., 2013; Doss et al., 2014). Polysomnography showed that individuals with central apnea and obstructive sleep apnea, display a decreased ventilatory drive in response to hypoxia and/or hypercapnia (Brunetti et al., 2021).

Although not located in the transmembrane domains of COX20, p.Glu9Lys is at a position of considerable importance for the stability and functionality of COX20. This variant is predicted (but shown conclusively) to disrupt protein structure causing the loss of an important hydrogen bond.

In family B, sequence variants in two genes seemingly underlie OSAS pathogenesis. While the precise function of the Protein tyrosine phosphatase domain-containing 1 (PTPDC1) protein is yet to be elucidated, its down regulation has been associated with phenotypes such as obesity (Zhu et al., 2020) and dementia (Wang et al., 2020). Functionally, PTPDC1 protein is involved in cilium assembly and degradation, directing fluid movement (motile cilia), sensing extracellular environment, and controlling signal transduction pathways (primary cilia). Thus, PTPDC1 depletion leads to an increase in cilia length, suggesting that it negatively regulates cilia length via increased anterograde transport (Lai et al., 2011).

The other variant present in family B is TMOD4, one of the four members of the Tropomodulin family, a group of proteins that bind to the pointed ends of actin filaments thereby stabilizing them and inhibiting filament elongation (Fischer & Fowler, 2003). *TMOD4* gene is highly expressed in skeletal muscle and heart (Cox & Zoghbi, 2000). The Inactivating biallelic pathogenic sequence variants in the *TMOD4* have been associated with myopathy (e.g., nemaline myopathy OMIM #609284) also reported in zebrafish-mutated model (Berger et al., 2014), suggesting that *TMOD4* mutations may lead to OSAS relevant myopathy, in the intercostal muscles and/or diaphragm.

An OSAS -like phenotype has previously been reported in cases with nemaline myopathy (Sasaki et al., 1997; Cook & Berkowitz, 2005). Sasaki *et al.* (1997), reported the results of polysomnography in two nemaline myopathy (NM) cases who had apnea or irregular chest movements with hypercapnia that occurred only during REM sleep. Cook and Berkowitz (2005) also described a milder form of NM in a child who presented at polysomnography, severe obstructive sleep apnea with an oxygen saturation nadir of 55% during REM sleep. Thus, these single reports are in line with the notion that loss of function in TMDO4 may play a role in muscle strength and may contribute to OSAS pathogenesis.

The wild-type amino acids in *COX20, PTPDC1* and *TMOD4* are highly conserved between species, which demonstrates its evolutionary relevance. Additionally, it is interesting to notice that the variants observed are rare in general population, demonstrating that it could be related to less adaptive subjects.

Noteworthy, no studies have previously identified possible genes related to mendelian forms of OSA and none of the above cited variants or other variants in the three genes reported herein have been identified in previous OSAS-GWAS studies. A search in GWAS catalog (https://www.ebi.ac.uk/gwas/genes) for variants within 150 Kb of the variants reported in the current study led to no results associated with sleep apnea. A possible reason is that SNPs of modest effect are missed as they do not reach a significance threshold (Eichler et al., 2010). Non-etheless, among the 41 OSA-associated GWAS variants, some trace back to genes involved in cytoskeletal structure and dynamic functionality, such as CDH4 (a calcium-dependent cell-cell adhesion glycoprotein that plays a role in muscle development), similarly to the *PTPDC1* and *TMDO4* genes mutated in Family B, which exhibit mainly structural functions important for muscle cell functionality. Furthermore, many of the GWAS OSA-associated variants display a pattern of participating in neuromuscular physiologic pathways and neurogenesis, such as RMST, CAMK1D and ATP2BA (Cade et al., 2016; Strausz et al., 2021). So far, most of the studies on OSA have focused on the effect of metabolic disorders such as obesity and environmental factors in the pathogenesis of OSA. However, the present finding of a syndromic form of OSA related to three genes with possible important roles in neuromuscular development and functionality, along with the existence of GWAS variants participating in similar pathways suggests that organic alterations and genetic susceptibility may play a pivotal role on the pathogenesis of OSA.

The limitations of our study should be acknowledged, such as lack of functional data, and limitations of exome sequencing for identification of non-coding and structural variants. Furthermore, the number of analyzed individuals from two families is limited and no segregation analysis could be performed in one of the two families, and the lack of replication in a second OSA family with variants in the same gene. Added to this, the results reported herein may only pertain to OSAS cases from Brazil.

In conclusion, the present study suggests that sequence variants in the *COX20*, *PTPDC1*, and *TMDO4* genes could be associated with OSAS phenotype in Brazilian families. The putative involvement of these genes in OSAS phenotype needs to be validated and expanded using more families of diverse ethnicity.

## Data Availability

The original contributions presented in the study are included in the article/Supplementary Material, further inquiries can be directed to the corresponding author.
