# Supplementary material for: Whole-exome identifies germline variants in families with obstructive sleep apnea syndrome
Source: Front Genet. 2023 May 9;14:1137817. doi: 10.3389/fgene.2023.1137817 (PMC10203477; doi:10.3389/fgene.2023.1137817)
Supplement: Supplementary file 1 [file Table1.DOCX]

SUPPLEMENTARY TABLE

Table 1 – Variants observed in all individuals with OSAS and absent in controls of the Family A.

| **Chr** | **Gene** | **Position** | **rs** | **c.DNA** | **Protein** | ***ACMG/AMP* classification** |
| --- | --- | --- | --- | --- | --- | --- |
| 1 | *COX20* | 244999041 | *rs946982087* | c.25G>A | p.Glu9Lys | VUS (PM2/PP2) |
| 9 | *NUP214* | 134049657 | *rs902499481* | c.3112A>G | p.Lys1037Glu | VUS (PM1/PM2) |
| 12 | *DNAH10* | 124333367 | *rs75173589* | c.5686G>A | p.Val1896Met | Likely benign (BP1/BS1) |
| 5 | *GPR98* | 90004717 | *rs202211640* | c.8815C>A | p.Pro2939Thr | VUS (PM2) |
| 16 | *WFDC1* | 84353166 | *rs145072849* | c.551G>A | p.Arg184His | Likely benign (BP1/BS1) |
| 9 | *TLE1* | 84205726 | *rs1333105043* | c.1823T>C | p.Leu608Pro | VUS (PM2/PP3) |
| 16 | *PKD1L2* | 81241175 | *rs79139155* | c.826C>T | p.Leu276Phe | VUS (PP3/BS1) |
| 15 | *VPS13C* | 62160874 | *rs77673743* | c.10847G>A | p.Gly3616Asp | Benign (BP1/BS1) |
| 15 | *TEX9* | 56657655 | *rs746610687* | c.7G>C | p.Gly3Arg | VUS (PM2/BP4) |
| 15 | *JMJD7* | 42127226 | *rs747062485* | c.277G>T | p.Ala93Ser | Benign (BP1/BS1) |
| 9 | *SHB* | 38068162 | *rs199974314* | c.481C>G | p.Leu161Val | Likely benign (BP1/BS1) |
| 11 | *MRGPRX4* | 18195609 | *rs146132319* | c.806C>T | p.Pro269Leu | Likely benign (BP1/BS2) |
| 11 | *MUC6* | 1031202 | *rs139011641* | c.541G>A | p.Gly181Arg | Likely benign (BP1/BS2) |

Table 2 – Variants observed in all individuals with OSAS and absent in controls of the Family B.

| **Chr** | **Gene** | **Position** | **rs** | **c.DNA** | **Protein** | ***ACMG/AMP* classification** |
| --- | --- | --- | --- | --- | --- | --- |
| 11 | *NLRP6* | 281073 | *rs946982087* | c.1339C>T | p.Arg447Cys | Likely benign (BP1/BS1) |
| 11 | *SLC22A18* | 2929502 | *rs143044180* | c.184G>T | p.Gly62Cys | Likely benign (BP2/BS2) |
| 19 | *ZNF433* | 12126348 | *rs202067147* | c.1229C>T | p.Thr410Met | Likely benign (BP1/BS3) |
| 18 | *POTEC* | 14542931 | *rs45554841* | c.215G>A | p.Cys72Tyr | Likely benign (BP1/BS1) |
| 15 | *PIF1* | 65108865 | *rs150356082* | c.1774C>T | p.Arg592Cys | Likely benign (BP1/BS1) |
| 9 | *PTPDC1* | 96857621 | *rs61743388* | c.477G>T | p.Trp159Cys | VUS (PM2/PP3) |
| 8 | *ZFPM2* | 106801092 | *rs202204708* | c.679A>G | p.Ile227Val | Benign (BP1/BS1) |
| 6 | *ZBTB24* | 109803084 | *rs147441359* | c.146G>A | p.Arg49Gln | Benign (BP1/BS1) |
| 5 | *LARS1* | 145503564 | *rs34823161* | c.3077A>G | p.Tyr1026Cys | Likely benign (BP1/BS1) |
| 1 | *TMOD4* | 151143043 | *rs141507115* | c.967G>A | p.Gly323Arg | VUS (PM2/PP3) |
