# Supplementary figures and images for: Whole-exome identifies germline variants in families with obstructive sleep apnea syndrome
Source: Front Genet. 2023 May 9;14:1137817. doi: 10.3389/fgene.2023.1137817 (PMC10203477; doi:10.3389/fgene.2023.1137817)

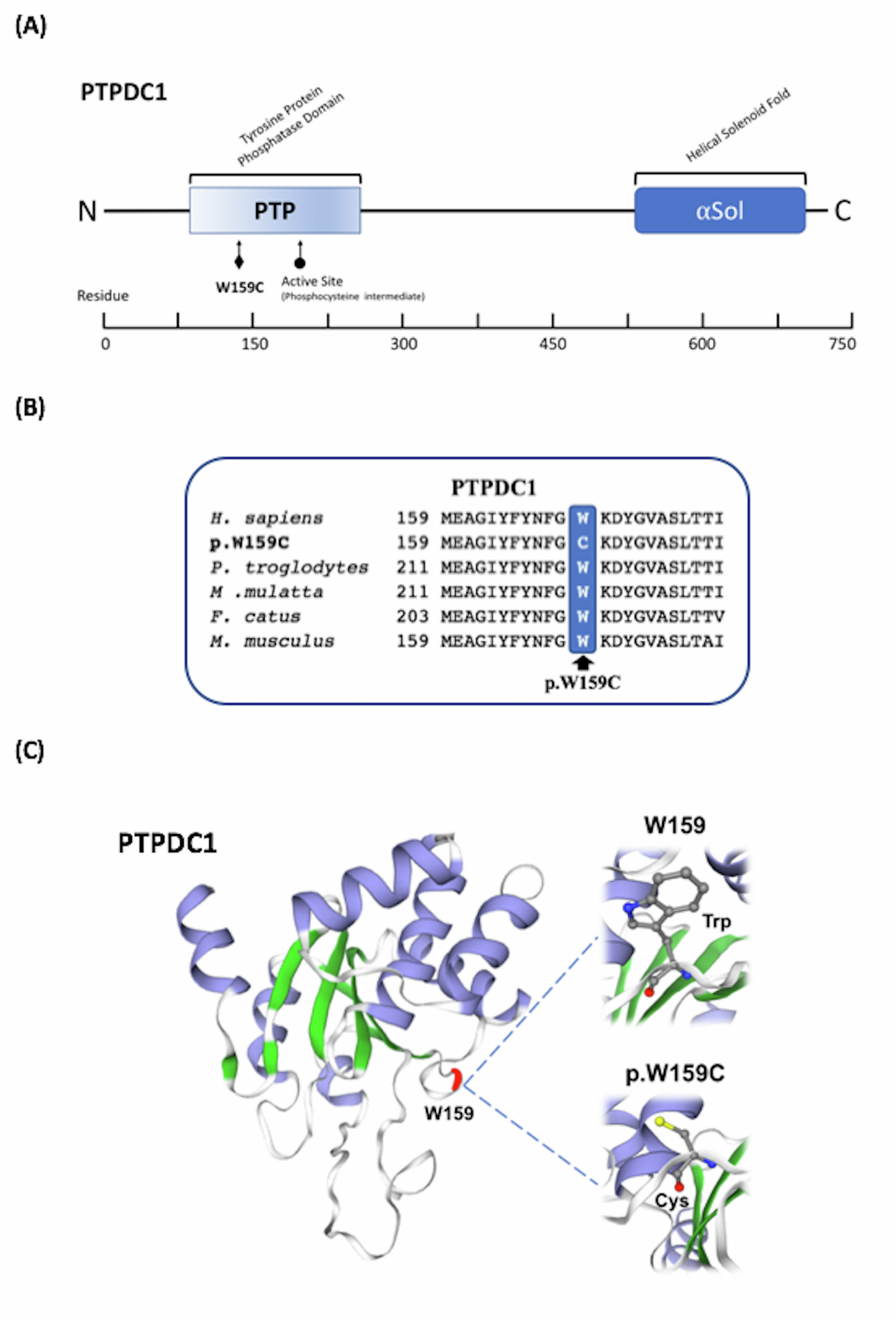

Supplement: Supplementary file 2 [file Image3.TIF]

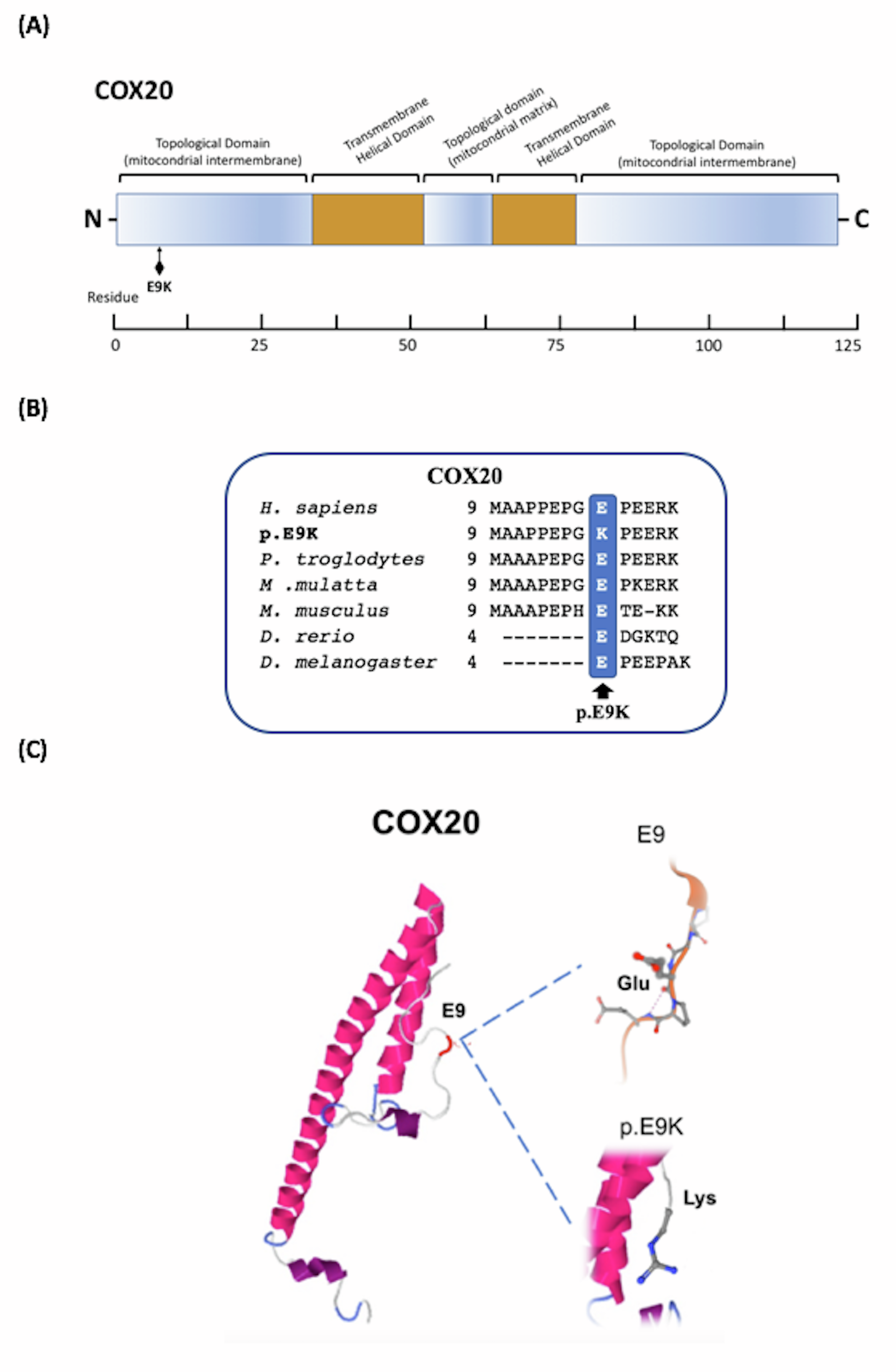

Supplement: Supplementary file 3 [file Image2.TIF]

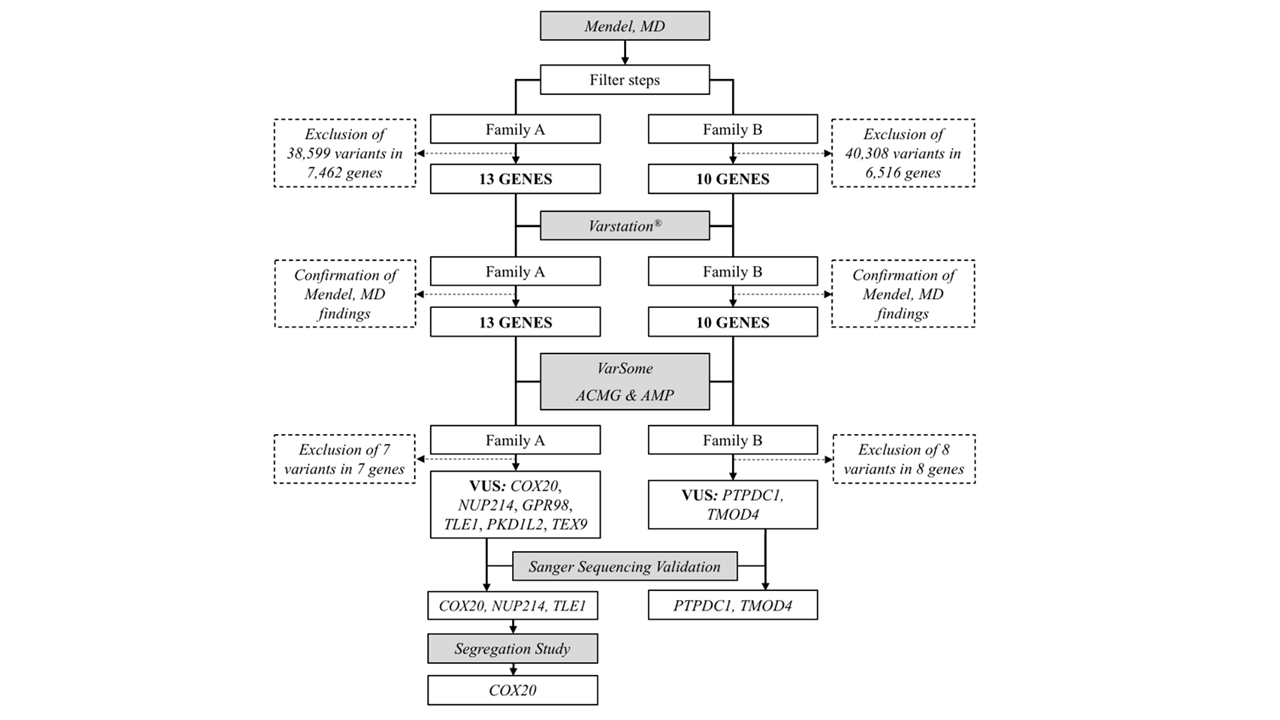

Supplement: Supplementary file 4 [file Image1.TIF]

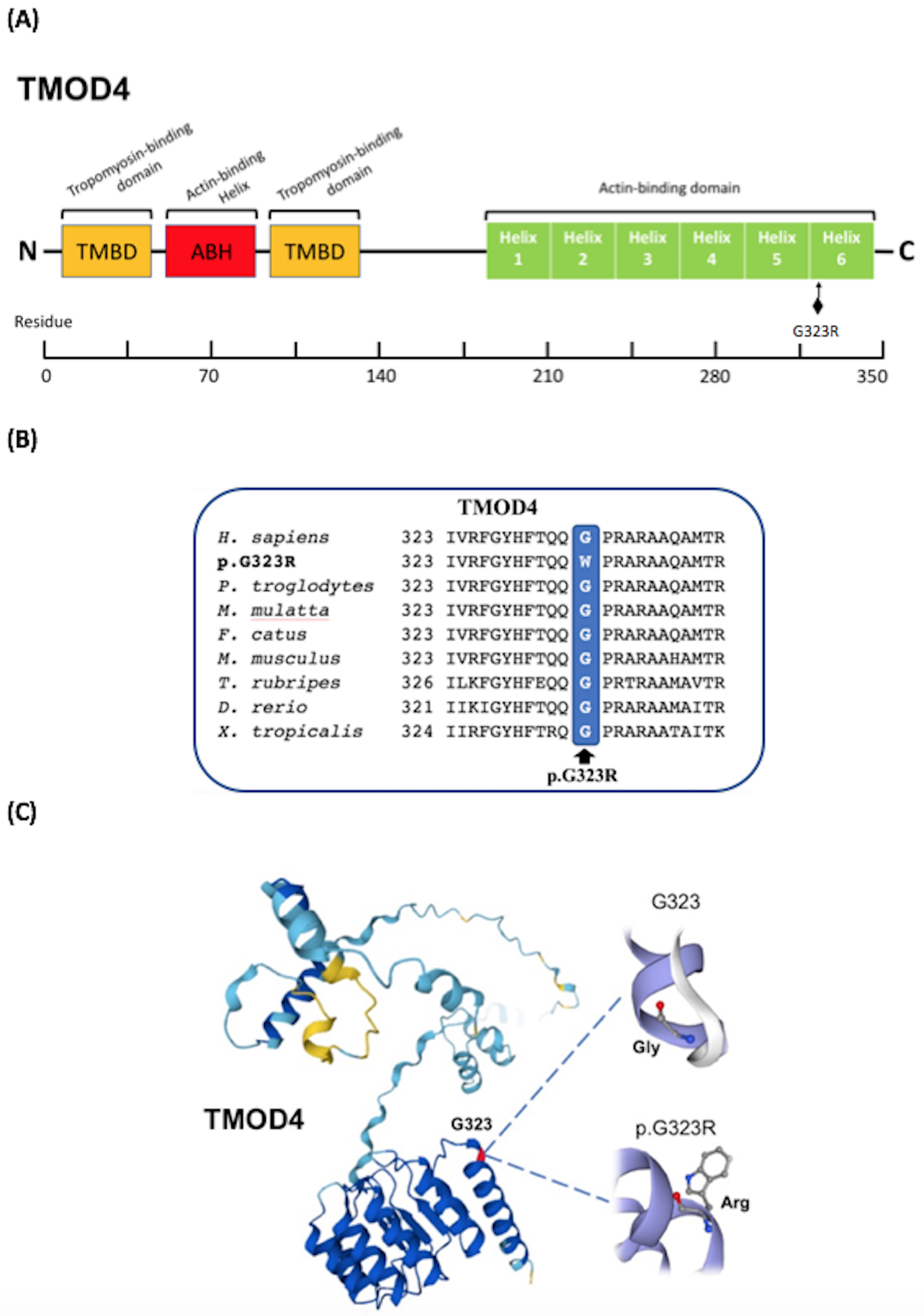

Supplement: Supplementary file 5 [file Image4.PNG]
